# Should we stop prescribing metoclopramide as a prokinetic drug in critically ill patients?

**DOI:** 10.1186/s13054-014-0502-4

**Published:** 2014-09-23

**Authors:** Y Gert van der Meer, Willem A Venhuizen, Daren K Heyland, Arthur RH van Zanten

**Affiliations:** Department of Hospital Pharmacy, Gelderse Vallei Hospital, Willy Brandtlaan 10, Ede, 6716 RP the Netherlands; Clinical Evaluation Research Unit, Angada 4, Kingston General Hospital, 76 Stuart Street, Kingston, ON K7L 2 V7 Canada; Department of Intensive Care, Gelderse Vallei Hospital, Willy Brandtlaan 10, Ede, 6716 RP the Netherlands

## Abstract

Regulatory agencies in North America and Europe recently re-evaluated the safety of metoclopramide. This re-evaluation resulted in recommendations and restrictions in order to minimise the risk of neurological and other adverse reactions associated with the use of metoclopramide. In the ICU, off-label prescription of metoclopramide is common. We have reviewed the evidence for safety, effectiveness and dosing of metoclopramide in critically ill patients. Furthermore, tachyphylaxis is addressed and alternatives are summarised. Finally, recommendations are presented not to abandon use of metoclopramide in ICU patients, because metoclopramide is considered effective in enhancing gastric emptying and facilitating early enteral nutrition.

## Introduction

In past years, regulatory bodies in North America have conducted reviews and issued warnings on metoclopramide. The US Food and Drug Administration has required boxed warnings and a maximum use of 12 weeks since 2009 [1], and a similar warning was issued in Canada in 2011 [2].

In December 2011 the French medicines regulatory agency L’Agence nationale de sécurité du médicament et des produits de santé requested a review of metoclopramide by the Committee on Medicinal Products for Human Use of the European Medicines Agency (EMA) because of continued safety and efficacy concerns. The Committee gave its opinion on 26 July 2013, which was followed by a re-examination requested by a manufacturer. This re-examination resulted in December 2013 in recommendations for and restrictions to the use of metoclopramide to minimise the risk of neurological and other adverse reactions associated with its use. Imposed restrictions included authorisation for short-term use (up to 5 days) only, lowering the maximum dose for adults to 30 mg/day or 0.5 mg/kg body weight/day – irrespective of the route of administration – and a ban on its use in patients with chronic conditions such as gastroparesis [3].

The formal EMA statement is as follows: ‘In order to minimise the risks of neurological and other adverse reactions, metoclopramide should only be prescribed for short-term use (up to 5 days). It should no longer be used in chronic conditions such as gastroparesis, dyspepsia and gastro-oesophageal reflux disease, nor as an adjunct in surgical and radiological procedures. In adults, metoclopramide remains indicated for prevention of post-operative nausea and vomiting (PONV), radiotherapy-induced nausea and vomiting and delayed (but not acute) chemotherapy-induced nausea and vomiting, and for symptomatic treatment of nausea and vomiting including that associated with acute migraine (where it may also be used to improve absorption of oral analgesics)’ [3]. No specific recommendations and restrictions for critically ill patients were provided.

## Why is there a safety issue with metoclopramide?

The EMA recommendations are based on the following reported side effects in non-ICU patients.

First, neurological adverse reactions, including extrapyramidal disorders, dyskinesia, dystonia, convulsion, hypertonia and tremor, can occur [4]. However, the absolute risk of neurological adverse reactions is poorly quantified. The reported occurrence of tardive dyskinesia ranges from <0.01 to 23% [5]. High occurrence is attributed to chronic use (months to years) of approximately 30 ± 10 mg metoclopramide/day [6] and concerns mostly older (60 ± 22 years) women [5]. Of an estimated 15.9 million metoclopramide prescriptions in the UK in the period 1967 to 1982, extrapyramidal symptoms were reported 479 times, representing an incidence of 0.003%. Of these reactions, 455 were dystonia–dyskinesia, 20 were parkinsonism and four were tardive dyskinesia [7]. Occurrence of akathisia is related to the rate of intravenous metoclopramide administration. After a bolus injection over 2 minutes, 24.7% of patients experienced akathisia, which was reduced to 5.8% in patients receiving the same dose in a 15-minute infusion [8]. Acute neurological adverse reactions in the ICU setting due to single use or short-term use of metoclopramide have not been reported in the literature to our knowledge, except for an anecdotal report on increased intracranial pressure for a short period after intravenous metoclopramide administration [9].

Cardiac adverse reactions, including shock, hypotension, cardiac arrest, tachycardia, bradycardia, hypertension, cardiorespiratory arrest and circulatory collapse, can also occur [4]. Evidence is solely based on case reports. The available case reports and case series (a total of 56 patients over a 44-year period) were recently summarised and discussed [10]. Of those, only three are critically ill patients. Moreover, the EMA assessment mentions ‘reports’, but does not refer to any specific publications in its references [4].

## The need to improve gastrointestinal motility in critically ill patients

Delayed gastric emptying is commonly encountered in the ICU and may be present in 50 to 60% of all ICU patients [11-13]. A recent retrospective analysis in ICUs from 21 countries demonstrated an enteral feed intolerance among 30.5% of patients after a median 3 days on enteral nutrition. Prokinetic drugs were administered in 37.9% of cases, primarily metoclopramide and erythromycin far less frequently [14]. Furthermore, enteral feed intolerance was associated with worse nutrition adequacy versus the tolerant (56% vs. 64%, *P* < 0.0001), fewer ventilator-free days (2.5 vs. 11.2, *P* < 0.0001), increased ICU stay (14.4 vs. 11.3 days, *P* < 0.0001), and increased mortality (30.8% vs. 26.2, *P* = 0.04) [14]. Studies such as the EDEN trial reporting on trophic feeding in contrast to full feeding have shown noninferior mortality in moderately obese and younger patients with acute respiratory failure, limiting the generalisability to other ICU patients [15]. In patients on the full feeding regimen there was a nonsignificant trend towards better long-term physical function [16]. Recent data suggest that providing at least 80% of prescribed amounts of protein and calories is associated with improved clinical outcomes and thus could be established as a quality indicator for ICU practice, particularly in high-risk patients [14].

## Present use of metoclopramide in critically ill patients

Metoclopramide is extensively used as an intravenous prokinetic drug to treat delayed gastric emptying and to facilitate early enteral feeding. However, metoclopramide is not officially registered for this specific indication in critically ill patients.

Off-label use of medications is common in medical practice. In many countries, off-label use is allowed when it is based on a firm scientific rationale and on sound medical evidence, preferably summarised in clinical practice guidelines [17-19]. However, the legal implications of clinical practice guidelines are not always clear [20]. Recently a study found that 79% of off-label prescriptions in primary care lack strong scientific evidence of efficacy [21]. A study in 37 ICUs in the USA in adult patients found that 36.2% of prescriptions were off-label, one-half of them with very little or no evidence to support their use [22].

The commonly used metoclopramide dosage in the ICU setting is 10 mg four times daily, although 10 mg three times daily is also used [11]. As tachyphylaxis to metoclopramide frequently occurs after a few days of treatment, intravenous erythromycin 200 mg twice daily can be added to enhance the prokinetic effects and decrease tachyphylaxis [23]. Early combination therapy of erythromycin and metoclopramide has been shown to be more effective than single administration of either drug [23].

Limited information is available on metoclopramide dosing in renal failure. It is usually recommended to reduce the dose by 50% when creatinine clearance is 10 to 50 ml/minute. Since the volume of distribution of metoclopramide is high, metoclopramide is only minimally removed by haemodialysis or peritoneal dialysis, and no supplemental dose is necessary after dialysis. During continuous renal replacement therapy, 50% of the normal dose can be prescribed [24-26].

## Prokinetics and early enteral nutrition

The benefits of early enteral feeding have been documented with a significant reduction in infectious complications and a trend towards a reduction in mortality in critically ill patients, as summarised in the Canadian Guidelines for Critical Care Nutrition [27,28]. Drugs to improve gastric emptying that may facilitate early enteral nutrition are therefore essential because gastric intolerance is frequently encountered during enteral nutritional support in the critically ill [13,14]. In several trials, prokinetics have been used prophylactically to improve feeding efficacy and prevent vomiting and ventilator-associated pneumonia in general ICU patients and in patients in the prone position during mechanical ventilation [29,30].

Since the restrictions imposed by the EMA and others apply to the approved indications of metoclopramide, how should these restrictions be translated into use in critically ill patients?

## Should we still consider the use of metoclopramide?

There appear to be three options regarding the use of metoclopramide: abandoning the use of metoclopramide and switching to available alternative(s); adjusting the dose to the recommended maximum daily dose of 30 mg; or waiving the imposed restrictions and continuing the present use of metoclopramide.

### Are there (available) alternatives to metoclopramide?

At present in many countries, intravenous erythromycin is the only available alternative to metoclopramide. Erythromycin is a macrolide antibiotic and, in addition to its antimicrobial activity, erythromycin is a motilin receptor agonist. As with metoclopramide, the use of erythromycin as a prokinetic drug to improve gastric emptying is off-label. Erythromycin is generally considered effective and some investigators suggest that erythromycin is even more effective than metoclopramide as a single prokinetic agent [13,31]. Commonly, 200 mg intravenous erythromycin twice daily is used, but doses of 70 mg might also be effective [32]. Use of erythromycin as a single agent may be hampered by the occurrence of tachyphylaxis within a few days and because of the concerns regarding its cardiac effects (QTc prolongation) and the potential emergence of antibiotic resistance [11,13].

Besides erythromycin, no other alternatives are currently available. Several new agents are being developed but have not yet been studied extensively in critically ill patients compared with metoclopramide and erythromycin. The following pharmacological agents are being tested or could be considered.

- Alvimopan and methylnaltrexone are opioid antagonists that reduce the hospital stay in postoperative patients and restore bowel function by antagonising the μ-receptor. Methylnaltrexone has been reported to improve feed intolerance in a single study, but alvimopan has not yet been studied in the critically ill [11,13].
- Ghrelin and its analogue RC-1139 are functionally related to motilin and accelerate gastric emptying [33,34]. In animal experiments, additional effects such as anticatabolic and growth hormone-like properties have been found. Studies in non-ICU patients on ghrelin suggest an increase in food intake and a positive effect on muscle strength [35,36]. Ghrelin has not yet been studied in the critically ill. RC-1139 has only proven to be a potent prokinetic in animal studies [11,13].
- Dexloxiglumide is a cholecystokinin-1 receptor antagonist. In critically ill patients, cholecystokinin is an important mediator of delayed gastric emptying. As a promoter of gastric emptying, dexloxiglumide has been found safe but not always effective in patients with functional dyspepsia and irritable bowel syndrome. Dexloxiglumide has not been tested in ICU patients [11,13].
- Tegaserod is a selective partial 5HT4 receptor agonist. Clinical improvement has been reported in an audit of critically ill patients with persistent feed intolerance [37,38]. The US Food and Drug Administration withdrew tegaserod from the market in 2007 for its use as short-term treatment of constipation-predominant irritable bowel syndrome, however, because of an increased risk of serious cardiovascular adverse events. This decision probably will preclude the use of tegaserod in critically ill patients [11,13,39].
- TD-8954 is another 5HT4 receptor agonist currently under investigation [40].
- Mitemcinal and ABT-229 are motilin agonists that are considered to have the prokinetic effects but not the antibiotic effects of erythromycin. However, the effects of ABT-229 have been disappointing, probably due to tachyphylaxis. Mitemcinal does accelerate gastric emptying in ambulant patients with gastroparesis, but has not been investigated in critically ill patients [11,13]. More recently, another motilin agonist (GSK 962040) has been tested in critically ill patients in phase I dosing studies and is presently in a phase II trial [41].
- Neostigmine is a cholinesterase inhibitor that increases acetylcholine concentrations at the neuromuscular junction, thereby enhancing the intestinal transit time. In a pilot study, a trend toward accelerated gastric emptying and improved feed tolerance was observed in critically ill patients [42]. However, an adequately powered study will be required to confirm these effects [11].

### Adjusting the dose of metoclopramide?

Adjusting the maximum daily dose to 30 mg or 10 mg three times daily according to the recommendations of the EMA should decrease the risk of neurological and other adverse reactions associated with metoclopramide [1]. However, evidence supporting the effectiveness of 10 mg three times daily in critically ill patients is scarce, since most studies among these patients investigated the effects of 10 mg every 6 hours [11]. One study did investigate metoclopramide 10 mg three times daily in the prevention of pneumonia in ICU patients who received enteral feeding through a nasogastric tube [43]. Enteral feeding via a nasogastric tube has been associated with an increased risk of ventilator-associated pneumonia, as impaired gastric emptying may promote migration of gastric microorganisms to the airways. By improving gastric emptying, metoclopramide is considered to decrease the risk of enteral feeding-associated pneumonia. Although metoclopramide three times daily did delay the onset of pneumonia in this study, this was shown not to be of clinical significance because the risk of pneumonia and mortality were not reduced [43]. Adjusting the metoclopramide dosage according to the recommendations of the EMA can thus not be recommended because there is a lack of evidence supporting this dosage regimen in critically ill patients. Efficacy has been shown in studies using 10 mg four times per day [23,31].

### Continue the use of metoclopramide as before?

As previously mentioned, intravenous metoclopramide is frequently used to treat delayed gastric emptying and to facilitate early enteral feeding, and its off-label use can be sufficiently supported by documented evidence. Use of metoclopramide in the ICU is applied only for short periods (days), limiting the risk of neurological adverse reactions since these are mainly associated with long-term use [3,4]. Moreover, the benefits of early enteral feeding may outweigh the occurrence of nonlife-threatening side effects.

## Tachyphylaxis of prokinetics

In critically ill patients the occurrence of tachyphylaxis might be more important for successful treatment than the occurrence of adverse reactions. Tachyphylaxis was addressed in two studies [23,31]. Treatment was successful when the gastric residue volume remained <250 ml with a nasogastric feeding rate ≥40 ml/hour. Treatment failure was defined as two or more high gastric residue volumes (≥250 ml) within the first 24 hours of treatment or any 6-hourly gastric residue volume ≥250 ml thereafter. The 7-day effectiveness of intravenous metoclopramide 10 mg four times daily, intravenous erythromycin 200 mg twice per day or combination therapy was investigated. After 24 hours, successful enteral feeding was achieved in 62% of patients treated with metoclopramide, in 87% of patients treated with erythromycin and in almost all (no percentage reported) patients treated with combination therapy. Over time, however, all treatments became less effective with treatment failure (that is, tachyphylaxis) occurring after 2 days, 3 days and 6.5 days for metoclopramide, erythromycin and combination therapy, respectively. At day 7 of treatment, successful feeding was achieved in 16% of patients treated with metoclopramide and in 31% of patients treated with erythromycin. No percentage is known for the combination treatment, but the rate of successful feeding was significantly higher than treatment with erythromycin as a single agent.

Desensitisation, downregulation and endocytosis of neurohumoral receptors have been proposed as mechanisms underlying the occurrence of tachyphylaxis [23,31]. Combination therapy is more effective because of the complementary actions of both prokinetics and the multiple mechanisms underlying delayed gastric emptying. The relevance of tachyphylaxis is still unknown, since combination therapy associated with delayed occurrence of tachyphylaxis did not result in improved survival or length of hospital stay. However, the power of the study might be insufficient to demonstrate significance [23,31].

## Recommendation and future perspectives

At present, the only available alternative to metoclopramide that has been extensively studied in critically ill patients is erythromycin, but both agents are commonly used as a combined strategy to improve gastric emptying and improve patient outcomes [11]. Some new prokinetic agents appear promising, but need testing in critically ill patients and therefore will not be available in the near future.

Weighing all of the pros and cons and considering all relevant aspects involved, we recommend to continue the off-label use of metoclopramide in critically ill patients and not to exceed a daily dose of 10 mg four times. Attention must be paid to the reported side effects and the occurrence of tachyphylaxis, since this might be of major importance for patient safety and successful treatment.

If intolerance to enteral feeding persists with use of metoclopramide, erythromycin should be added for another 24 to 48 hours. If this combination does not work, in our opinion it is probably better to stop both agents and move to another route of feeding such as postpyloric feeding. In any case, we recommend using the combination only during a maximum of 7 days to limit side effects related to prolonged exposure and for reasons of tachyphylaxis. In cases of renal failure, the dose of metoclopramide should be adjusted.

We hope that new prokinetics will come to the market with high effectivity, limited or absent tachyphylaxis and no serious side effects.

## Abbreviation

EMA: European medicines agency
